# Crack Detection on a Retaining Wall with an Innovative, Ensemble Learning Method in a Dynamic Imaging System

**DOI:** 10.3390/s19214784

**Published:** 2019-11-03

**Authors:** Chern-Sheng Lin, Shih-Hua Chen, Che-Ming Chang, Tsu-Wang Shen

**Affiliations:** 1 Department of Automatic Control Engineering, Feng Chia University, Taichung 407, Taiwan; chenshihhua0118@gmail.com (S.-H.C.); TWSHEN@fcu.edu.tw (T.-W.S.); 2 Ph.D. Program of Electrical and Communications Engineering, Feng Chia University, Taichung 407, Taiwan; hcchang@fcuoa.fcu.edu.tw

**Keywords:** unmanned vehicle, innovative ensemble learning, cascade classifier, edge feature comparisons

## Abstract

In this study, an innovative, ensemble learning method in a dynamic imaging system of an unmanned vehicle is presented. The feasibility of the system was tested in the crack detection of a retaining wall in a climbing area or a mountain road. The unmanned vehicle can provide a lightweight and remote cruise routine with a Geographic Information System sensor, a Gyro sensor, and a charge-coupled device camera. The crack was the target to be tested, and the retaining wall was patrolled through the drone flight path setting, and then the horizontal image was instantly returned by using the wireless transmission of the system. That is based on the cascade classifier, and the feature comparison classifier was designed further, and then the machine vision correlation algorithm was used to analyze the target type information. First, the system collects the target image and background to establish the samples database, and then uses the Local Binary Patterns feature extraction algorithm to extract the feature values for classification. When the first stage classification is completed, the classification results are target features, and edge feature comparisons. The innovative ensemble learning classifier was used to analyze the image and determine the location of the crack for risk assessment.

## 1. Introduction

The remote cruise routine of an unmanned vehicle was developed and implemented for searching optimal traveling paths, commonly referred to as traveling salesman problem (TSP) and the shortest path [1,2,3,4]. Osch made the remote operated service robot for the home environment and simple basic tasks, and developed home care tasks by remote operation. These designs can be used for developing service robots for security, building maintenance, etc. [5,6]. Many implementation problems can be solved by the robot education platform [7]. The remote cruise control movement track of an unmanned vehicle considers the starting point and end point, and the movement path, as well as actual speed and acceleration difference are calculated. The path may contain different trajectories, which represent an actual path, and a vehicle trajectory can be generated by using the shortest path [8,9]. The monitoring process of an unmanned vehicle applying machine vision techniques is mostly based on methods using three-dimensional imaging systems, or two-dimensional systems with the limitation of monitoring under various conditions [10].

A computer vision-based method is often used to automatically detect cracks and record crack information, such as the shape, location, and width [11].

This study was mainly to develop an integrated learning classifier for a dynamic imaging system of an unmanned vehicle. This unmanned vehicle can provide a lightweight and remote cruise routine with a Geographic Information System (GIS) sensor [12,13,14], a Gyro sensor, and a charge-coupled device (CCD) camera for the imaging task of the retaining wall in a climbing area or a mountain road. The retaining wall is patrolled through the drone flight path setting, and then the image is instantly returned by using the wireless transmission of the system. Based on the cascaded classifier created, the target position to be detected is detected first. If the detection target is successfully classified, then the feature comparison classifier is selected [15]. Detailed information on the classification target is sent, and finally, the system is actually operated to detect the cracks of retaining wall.

Differently to the existing crack-detection techniques, this study changes the sample weight with the number of iterations. The sample classification result of the previous layer will adjust the weight for the next time; for the sample of the previous classification error, a larger weight value will be given again. The samples that have been classified correctly reduce the weighting value. And the results of enhanced identification can be obtained for samples that are difficult to classify. This study has good stability even if the target is obscured and the light source changes.

In the experiments, the remote cruise routine was set to the tested area of retaining wall, and the horizontal image was instantly transmitted back to the computer during the imaging process [16,17,18,19]. When the computer received the image, the system classifier was used for detection. When the position of the cracks was classified, the coordinate data was obtained. The feature comparison classifier was used to classify the different postures of the retaining wall with a canny operator, local binary patterns (LBP), and the ensemble learning method.

## 2. Canny Detection

Generally, the edge is a significant change in the brightness of the set of pixels in the image; that is, the change of 1 to 0, and has discontinuity, which is also an important feature that image segmentation often depends on. In this study, Canny detection was used, because Canny detection is an ideal balance between the detection effect and the amount of computation compared to other edge-detection algorithms [20,21].

The steps for Canny detection are as follows:

(1) The edge detection operation is mainly based on the first-order and second-order derivatives of the image intensity, but the derivative is very sensitive to noise, so the Gaussian filter equation (1) is used to smooth the image in order to reduce noise, to avoid the impact of noise on the test results.
  (1) $$H_{i,j}=\frac{1}{2\pi \sigma^{2}}e^{-\frac{{\left(i-k-1\right)}^{2}+{\left(j-k-1\right)}^{2}}{2\sigma^{2}}},$$
  where $H_{i,j}$ is a Gaussian function, taking k=1 and σ=1, and then a 3 × 3 Gaussian filter convolution kernel is obtained:
  (2) $$H=\left[\begin{array}{ccc} \frac{1}{2\pi }e^{-1} & \frac{1}{2\pi }e^{-0.5} & \frac{1}{2\pi }e^{-1}\\ \frac{1}{2\pi }e^{-0.5} & \frac{1}{2\pi } & \frac{1}{2\pi }e^{-0.5}\\ \frac{1}{2\pi }e^{-1} & \frac{1}{2\pi }e^{-0.5} & \frac{1}{2\pi }e^{-1} \end{array}\right]=\left[\begin{array}{ccc} 0.0585 & 0.0965 & 0.0585\\ 0.0965 & 0.1529 & 0.0965\\ 0.0585 & 0.0965 & 0.0585 \end{array}\right].$$
(2) Calculate the gradient relationship after image smoothing:
  First, take the first-order differential convolution template, as in Equation (3):
  (3) $$H_{x}=\left|\begin{array}{cc} -1 & -1\\ 1 & 1 \end{array}\right|,\;H_{y}=\left|\begin{array}{cc} 1 & -1\\ 1 & -1 \end{array}\right|$$
  Calculate the partial derivatives of *x* and *y*, as in Equations (4) and (5), and then calculate the edge strength and edge direction of the image, which are respectively given by Equations (6) and (7):
  (4) $$G_{x}\left(x,y\right)=f\left(x,y\right)*H_{x}\left(x,y\right)$$
  (5) $$G_{y}\left(x,y\right)=f\left(x,y\right)*H_{y}\left(x,y\right)$$
  (6) $$G\left(x,y\right)=\sqrt{G_{x}{}^{2}\left(x,y\right)+G_{y}{}^{2}\left(x,y\right)}$$
  (7) $$\theta \left(x,y\right)=\tan^{-1}\frac{G_{x}\left(x,y\right)}{G_{y}\left(x,y\right)},$$
  where f(x,y) is the original image after Gaussian filtering convolution, G(x,y) is the edge intensity, and θ(x,y) is the edge direction.
(3) The larger the value of the element in the image gradient magnitude matrix, the larger the gradient value of the point, but it cannot be said that the point is the edge. The non-maximum value suppression is used to detect along the gradient direction. The center pixel G(x,y) is compared with two pixels along the gradient line; if the gradient value of G(x,y) is not larger than the gradient values of two adjacent pixels along the gradient line, it can be judged that the pixel is not an edge; then, let G(x,y)=0, which eliminates areas with large gradients near the edges.
(4) In order to obtain a characteristic and closed edge with a double threshold, when the image gradient is greater than the high threshold, an image with a small number of false edges and sharp features can be obtained, but the image edges may not be closed, so set a low threshold and find the point at the edge of the edge that meets the low threshold to complete the image edge.

## 3. Ensemble Learning

Ensemble learning is the process of bringing together a number of different classifiers, each with different weights, and combining the different weighted classifier results as the final classifier.

### 3.1. LBP Feature

Local binary patterns (LBP) mark the pixels of an image by thresholding each pixel’s neighborhood using the value of the center pixel, and treat the result as a binary number, as in Equations (8) and (9):

(8) $$\mathrm{LBP}\left(x_{c},y_{c}\right)=\sum_{p=0}^{p-1}2^{p}s\left(i_{p}-i_{c}\right)$$

(9) $$s(x)=\{\begin{array}{cc} 1\; & if\;x\geq 0\\ 0 & else \end{array},$$

where $\mathrm{LBP}\left(x_{c},y_{c}\right)$ is the LBP value $\left(x_{c},y_{c}\right)$ of the image as the coordinates of the central pixel; p is the p-th pixel of the coordinate neighborhood of the central pixel; and $i_{p}$ is the gray value of the neighborhood pixel $i_{c}$ for the gray value of the center pixel s(x), a sign function.

**Figure 1:**
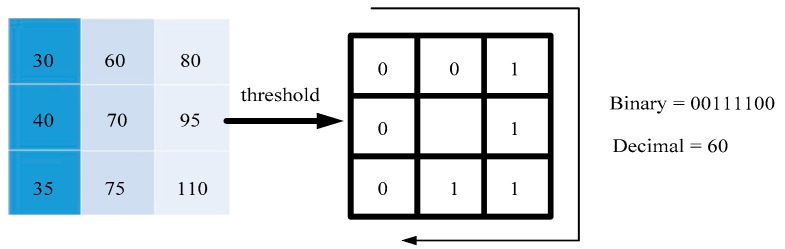
Local binary patterns (LBP) conversion.

Within a 3 × 3 window, the pixel at the center of the window is the threshold. If it is greater than the threshold, it is marked as 1, and vice versa. Thus, eight points in the 3 × 3 neighborhood can be compared to generate an 8-bit binary value, and the binary value is converted into decimal; that is, the LBP value of the central pixel of the window is obtained, as shown in Figure 1. Use this value to reflect the texture information of the region as the feature value [22,23].

In Figure 1, there are $2^{8}$ kinds of binary cases in the 3 × 3 window. As the number of neighbors increases, the number of binary patterns will increase rapidly. To solve the problem of too many binary patterns, a kind of problem is adopted. The uniform pattern, in most LBP features, can only have up to two jumps from 1 to 0 or 0 to 1, such as 00000000 (0 total transitions), 00001111 (1 total transition), or 01001111 (a total of two hops). In the two hops, the uniform pattern is classified into the same category, except for the mode other than the uniform pattern, which is classified as another class, called a mixed mode class, such as 10010111 (four jumps in total).

The uniform pattern formula is as follows:

(10) Uniform LBP=P(P−1)+2.

Among them, P represents the number in the neighborhood. It can be seen from the calculation result of Equation (11) that the binary mode can be reduced from the original 256 classes to 58 classes through the equivalent mode, plus the mixed mode class. If all of the binary numbers of the LBP operator are obtained in LBP equivalent mode, it can effectively reduce the dimension of the binary pattern. The LBP features can be divided into 59 categories, in addition to reducing the amount of computation, and reducing the impact of high-frequency noise.

### 3.2. AdaBoost Cascading Classifier

The AdaBoost algorithm constructs a classifier that customizes multiple classifiers, and each classifier has a high recognition rate. The AdaBoost algorithm is applied on the 59 classes of LBPs. Only the classifiers in the classifier are detected. When the image is mostly background, it can be screened out early, which can significantly reduce the number of calculations [24].

(1) First, define the representation of the number of samples, such as is in Equation (11):
  (11) $$T=\left\{\left(x_{1},y_{1}\right),\ldots ,\left(x_{i},y_{i}\right),\ldots ,\left(x_{N},x_{N}\right)\right\},$$
  where *T* represents the training database, *x* represents the sample, *y* is the set of tokens {−1, 1}, *x_i* represents the *i*-th sample in the training library, and *N* represents the number of samples.
(2) Initialize the weight distribution of the training database. Each training sample is initially given the same weight:
  (12) $$w_{1i}=\frac{1}{N},\;i=1,2,\ldots ,N,$$
  where $w_{1i}$ refers to the initial weight of the sample and *N* is the total number of samples.
(3) Use the weight distribution to get the basic classifier:
  (13) $$G_{m}\left(x\right):\;x\to \left\{-1,+1\right\}$$
  where *m* is the number of iterations, and the iteratively classified result is +1 if the target is the object, and negative sample 1.
(4) After the number of iterations m, select the classifier from:
  - Calculate the classification error rate of $G_{m}\left(x\right)$ on the training samples:
    (14) $$\varepsilon_{m}=\sum_{i=1}^{N}w_{m}\left(i\right)\left(G_{j}\left(x_{i}\right)\neq y_{i}\right)$$
    Equation (14) indicates that if the sample result value classified by the j-th classifier after the *m*-th iteration is not equal to $y_{i}$, it means that the classification error is performed, so the sample weight $w_{m}\left(i\right)$ of the classification error is accumulated, which is $\varepsilon_{m}$ the value.
  - The smallest cumulative error value $\varepsilon_{m}$ is selected from all *j* classifiers, which is the best classifier for the *m*-th iteration at that time, and the formula is as shown in Equation (15):
    (15) $$G_{t}=G_{j}\;with\;\min\left(\varepsilon_{m}\right)$$
  - Calculate the weight of the best weak classifier defined by Equation (15):
    (16) $$\alpha_{t}=\frac{1}{2}\ln\left(\frac{1-\varepsilon_{m}}{\varepsilon_{m}}\right)$$
    In Equation (16), εm≤12, $\alpha_{t}\geq 0$; then, $\alpha_{t}$ will increase as the *ε_m_* decreases, because the smaller the classification error rate, the more important the classifier is, so Equation (16) can be used. The smaller the error value, the larger the weight value.
  - Update the weight of all training samples:
    (17) $$w_{i}^{m+1}=\frac{w_{m}\left(i\right)\exp\left(-\alpha_{t}y_{i}G_{m}\left(x_{i}\right)\right)}{Z_{m}}$$
    Among them, $Z_{m}$ is the new sample weight, and the re-value is normalized between 0 and 1.
    (18) $$-\alpha_{t}y_{i}G_{m}\left(x_{i}\right)\{\begin{array}{ll} <0, & y_{i}=G_{m}\left(x_{i}\right)\\ >0, & y_{i}\neq G_{m}\left(x_{i}\right) \end{array},$$
    where $G_{m}\left(x_{i}\right)$ is a positive sample in the classifier.
    In Equations (17) and (18), it is shown that the weight value of the correctly classified sample is reduced after normalization, and because of Equation (16), the sample weight of the error is increased, so the weight at this time is normalized. The latter value will increase, and in this way, it can focus on the more difficult to distinguish samples.
(5) After *m* iterations, *m* classifiers are obtained, and the weights are added according to the importance of each classifier, and finally, the strong classifier H(x) is obtained:
  (19) $$\mathrm{Hi}\left(x\right)=\mathrm{sign}\left(\sum_{m=1}^{m}\alpha_{m}h_{m}\left(x\right)\right)$$

From the AdaBoost algorithm, the sample weight is mainly changed with the number of iterations. The sample classification result of the upper layer will adjust the weight of the next time; for the sample of the previous classification error, a larger weight value is re-given. The sample that has been classified correctly reduces the weight value of the sample, and can focus on the samples that are difficult to classify. At the end of the study, multiple classifiers of strong classifier H_1_–H_i_ can be obtained [25,26].

### 3.3. Feature Comparators

**Figure 2:**
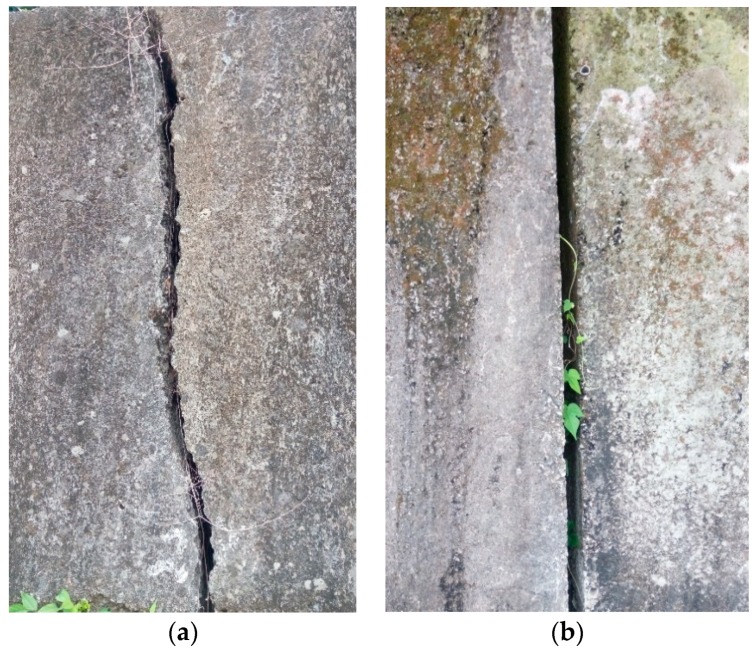
(**a**) The shape of the crack is different and the danger rate is different. (**b**) A joint (not a crack).

When the target itself can be distinguished if there are more subtle features, it is like the analysis of the dynamic images of the retaining wall, as shown in Figure 2.

A sample image was prepared in advance, and the sample contained data that distinguished the important features of the target, and each image was presented in the same size. The feature extraction of the image used the features extracted by Canny edge detection, and then each image was extracted. The edge feature created the final repository.

Using the gradient information obtained by edge detection for comparison analysis, first define the $P_{i}$ coordinate as the edge point of the template and $P_{i}={\left(X_{i},Y_{i}\right)}^{T}$, which is the distance between the center of gravity and the edge of the template image, and define the phase The gradient direction vector of the corresponding gradient feature coordinate point $P_{i}$ is $d_{i}={\left(t_{i},u_{i}\right)}^{T},i=1,2,\ldots n$, i represents the coordinate point of the sequential search.

Define the gradient direction vector of the region coordinate point in the image to be tested as (r, c) as $e_{i}={\left(V_{r_{i},c_{i}},W_{r_{i},c_{i}}\right)}^{T}$, and Equation (20) is the target to be tested. The similarity calculation between samples:

(20) $$s1\left(x,y\right)=\frac{1}{n}\sum_{i=1}^{n}d_{i}\cdot e_{i}=\frac{1}{n}\sum_{i=1}^{n}t_{i}\cdot V_{x+r_{i},y+c_{i}}+u_{i}\cdot W_{x+r_{i},y+c_{i}}$$

where s1(x,y) is the result of the similarity calculation result, n is the number of edge coordinate points in the image, and the inner product of the sample and the gradient vector of the target to be measured is added to the inner product of all coordinate points of the image. The similarity value of the coordinate point (x,y) can be obtained.

Then, Equation (20) is normalized so that the value can be borrowed between −1 and 1, as shown in Equation (21):

(21) $$s2\left(x,y\right)=\frac{1}{n}\sum_{i=1}^{n}\frac{d_{i}\cdot e_{i}}{\left\|d_{i}\|\|e_{i}\right\|}=\frac{1}{n}\sum_{i=1}^{n}\frac{t_{i}\cdot V_{x+r_{i},y+c_{i}}+u_{i}\cdot W_{x+r_{i},y+c_{i}}}{\sqrt{t_{i}{}^{2}+u_{i}{}^{2}}\sqrt{V_{x+r_{i},y+c_{i}}^{2}+W_{x+r_{i},y+c_{i}}^{2}}},$$

where s2(x,y) is the normalized similarity of the result value. If the value is normalized by Equation (21), all the values are between −1 and 1. When the calculated similarity s = 1, it means that the target and the sample are perfectly matched, and there is a clear range to define the threshold of similarity, and the noise similarity or other factors of noise similarity. When the value is scaled to between −1 and 1, it will be a small value, which will not affect the comparison of the similarity between the target and the sample. Therefore, the similarity calculation of Equation (21) is for the target. Factors such as shadowing and light source changes have good stability [27].

## 4. Experimental Results

**Figure 3:**
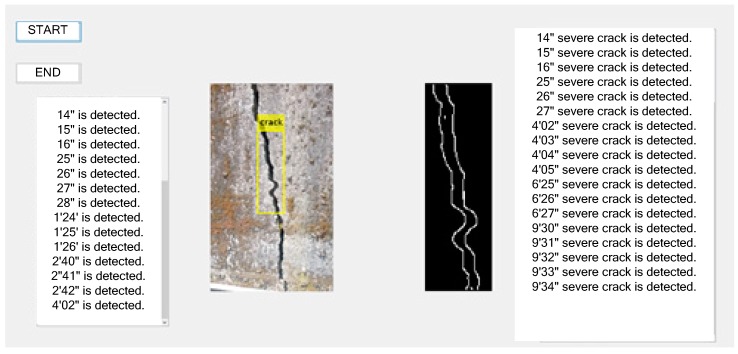
Operation interface for crack detection of retaining wall.

The drone model was the MAVIC 2 Pro. When the drone loses its remote control or the battery is low, it can use the automatic return function to fly in the direction selected by the pointer. When the drone flies to the mountain area for imaging the retaining wall, the user can see the flight process. The MAVIC 2 Pro features an omnidirectional sensing system that provides effective detection and sensing in most flights. It has a binocular vision sensor in front and rear, with a front detectable range of 20–40 m and a rear detectable range of up to 16–32 m, and monocular vision sensors on the left and right sides, which can detect obstacles to assist obstacle avoidance. The binocular vision sensor and infrared distance sensor below, can achieve accurate hovering of 50 m height, and ensure landing safety through terrain detection [28,29,30,31]. When using the pointing flight function, such as encountering obstacles during flight, it can dodge or hover to improve flight safety. For the creation of cascading classifiers to classify the target locations and the environmental results of the system for image processing (Figure 3), there are quite good experimental results, and the location of the cracks can be successfully and completely classified. Under ideal conditions, the effective distance of the remote control technology can reach 8 km. If the obstacle is not included in the middle, it can reach 10 km. Under normal conditions, the image quality is HD (720p). If it is close to the distance, there is no interference. In case the image quality, it can be Full HD (1080p).

### 4.1. Training Classifier

For the training classifier, three different sample sizes were used to identify the same 100 test images of cracks. When selecting a negative sample, in general, any non-cracked image can be selected as a negative sample, but a more reasonable approach would be to consider the actual application. From the data in Table 1, it can be seen that with the increase of the number of positive and negative samples, the accuracy rate is obviously improved [15,16,17,18]. The image that cannot be discriminated is an image that cannot be classified and discriminated in 100 test images. The correct result indicates that the correct crack position is classified among the number of results that can be classified; in the unrecognizable image, most of them are analyzed. It is a type of crack that is relatively rare in positive samples, so the subsequently increased samples are complemented by those types of cracks, so it can be seen from the data that the number that cannot be discriminated is reduced.

**Figure 4:**
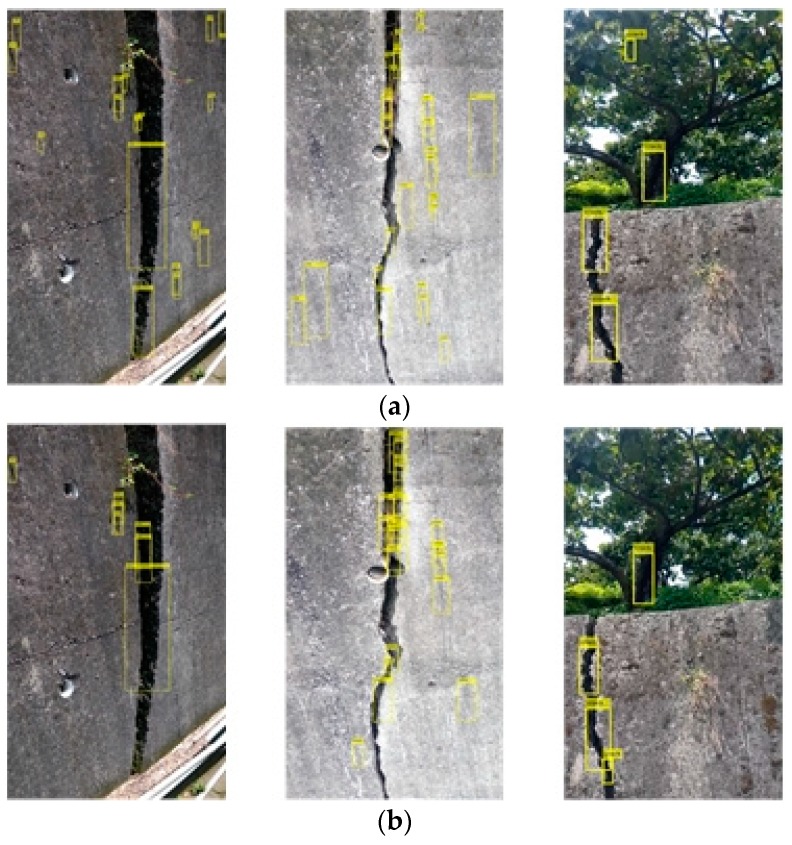

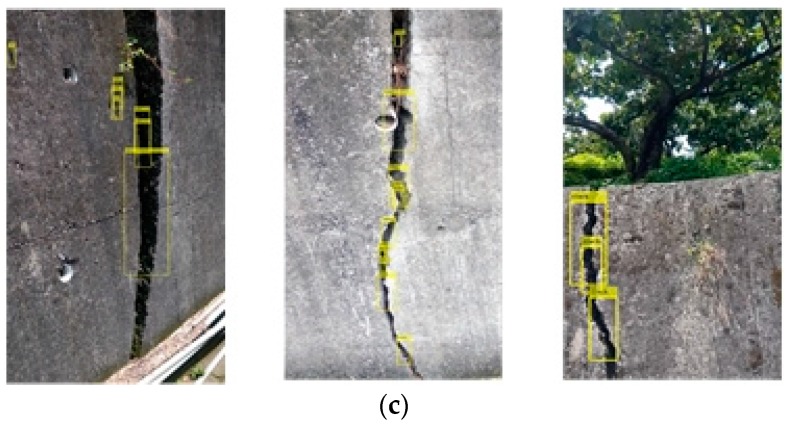
Classification results of dangerous cracks by cascade classifier with positive sample numbers (**a**) 200, (**b**) 500, and (**c**) 1000.

Figure 4 shows the classification result of the actual test crack image by the cascade classifier. Figure 4a–c respectively, show the training samples of the positive sample numbers 200, 500, and 1000 in Table 1, and the number of samples was only 200. When the image is displayed, the position of the crack can be basically classified, but the surrounding background is also misclassified, and the classification of the training of 500 and 1000 samples significantly improves the background misjudgment in the test image. The third one will classify the shadows of the trees, so when the second and third training samples are followed, the image of the shaded trees is increased to the negative sample, so from Figure 4b,c, one can see a significant improvement, and in the first test image in Figure 4c, we can see that there is a misclassification classification: the classifier regards the weeds growing on the retaining wall as cracks. When creating a classifier, the system must add multiple images like this to the negative sample. The bounding boxes show whether there is a misjudgment. For example, the classifier treats the weeds growing on the retaining wall as cracks. When creating the classifier, it is necessary to add multiple images similar to this to the negative samples. For example, there is a water pipe under the image of the retaining wall to block the crack of the retaining wall. When the classifier is designed well, it can only distinguish the upper expansion joint from the classification result, and it will not be misjudged because of the lower pipeline.

**Figure 5:**
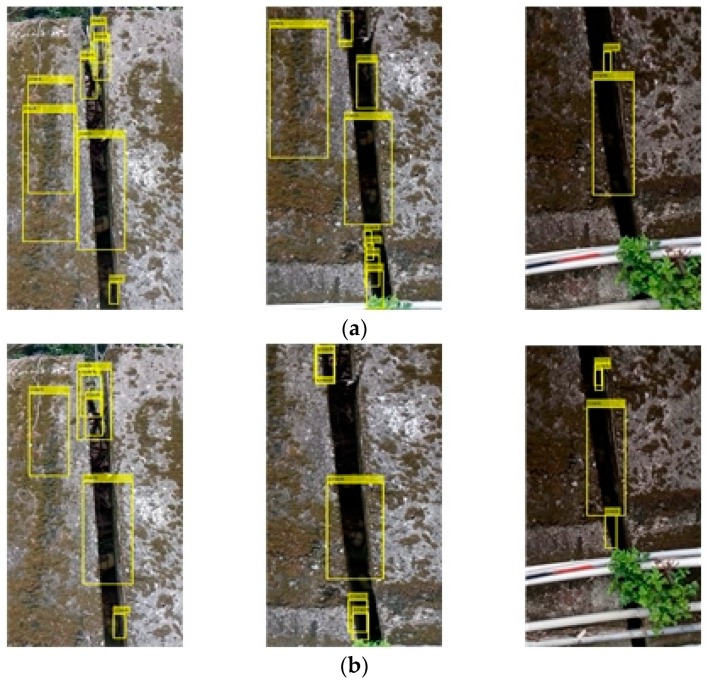

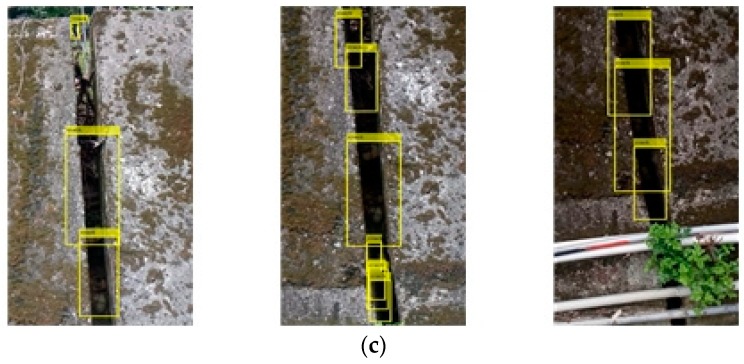
Classification results of cascaded classifiers for joint with positive sample numbers (**a**) 200, (**b**) 500, and (**c**) 1000.

In Figure 5a–c, the cascading classifier trained in the sample data table in Table 1 classifies the results of joint classification, and they can be discriminated from the classification even if the crack is non-hazardous. The cracks will still have classification results through the cascade classifier, so the subsequent feature comparison classifier will be needed. In the third test image retaining wall, there is a water pipe to block the crack of the retaining wall. From the classification results, it is also possible to judge only the expansion joint above, and it is not misjudged by the lower pipeline.

### 4.2. Crack Feature Extraction

**Figure 6:**
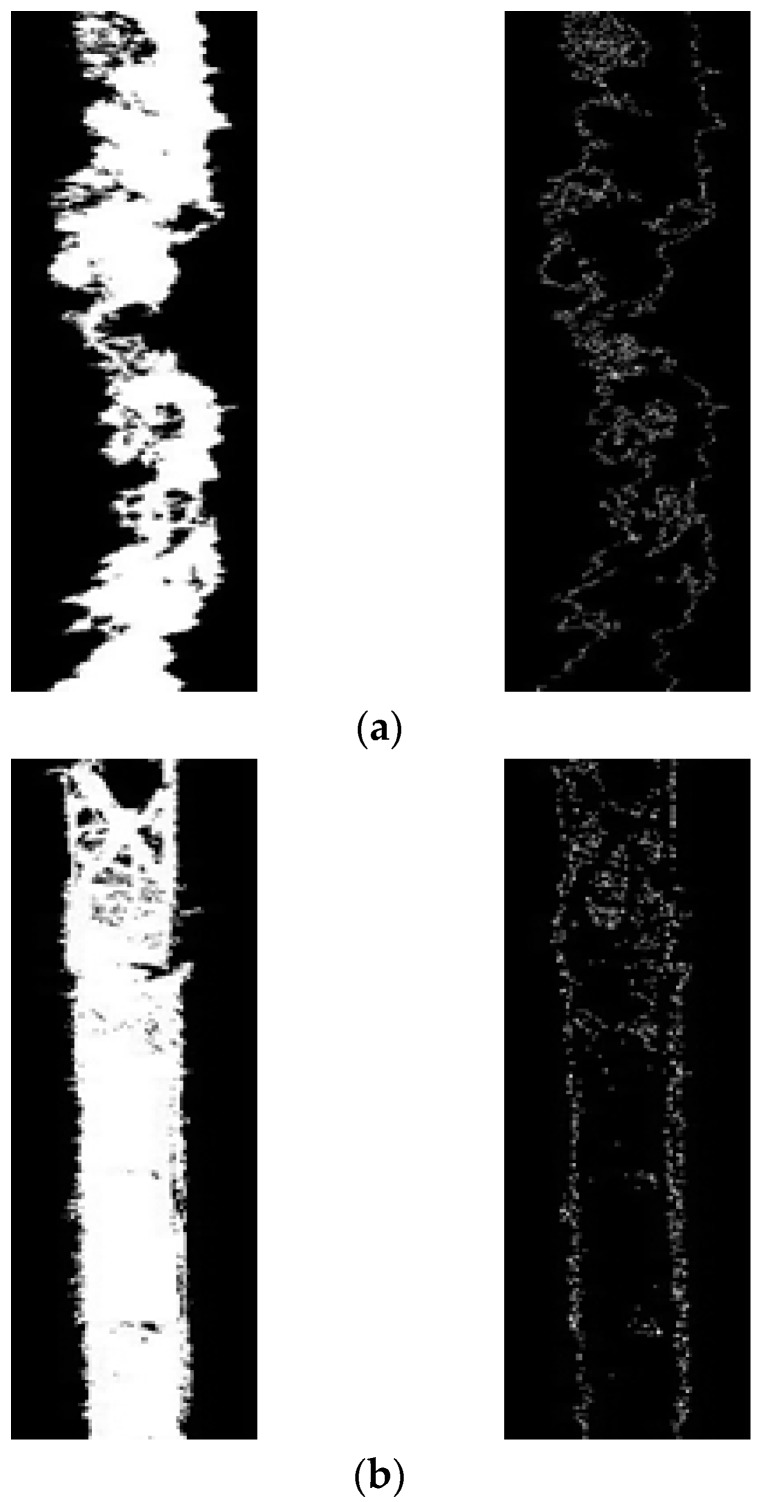
(**a**) Hazardous crack characteristics; (**b**) joint feature image.

The test results are further analyzed in Figure 4 and compared with the image of Figure 5a—non-dangerous cracks. First, the image is pre-processed by grayscale, binarization, etc., followed by Canny edge detection, as shown in Figure 6. From the binarization of the two images of Figure 6a,b, it is possible to clearly judge the difference between the edges of the two, so it is judged whether it is a dangerous crack by this edge.

**Figure 7:**
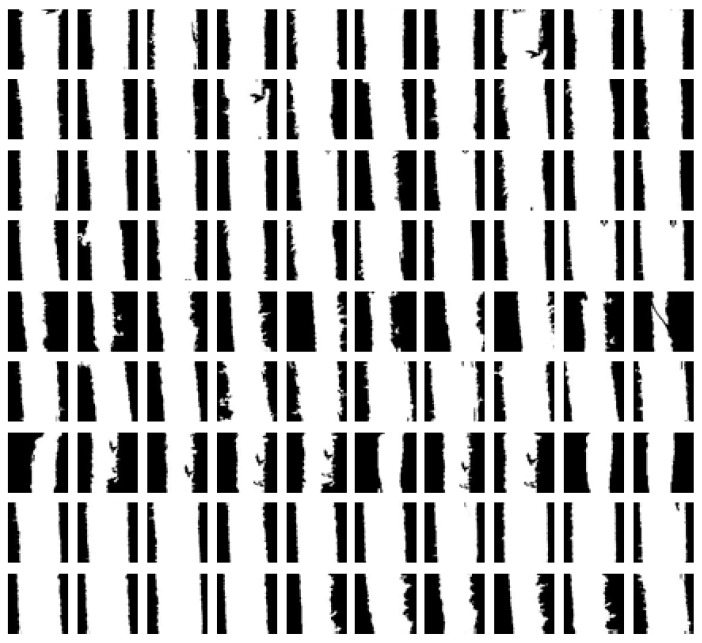
Joint sample database.

The sample in Figure 7 is a joint selected from the 1000 positive samples of the result of Figure 5. After image pre-processing and filtering processing, the sample size is also the same, 100 × 100, and in Figure 7, it is obvious that most of the joint edges are relatively flat, so it is obvious that the dangerous cracks and joints are correctly classified.

**Figure 8:**
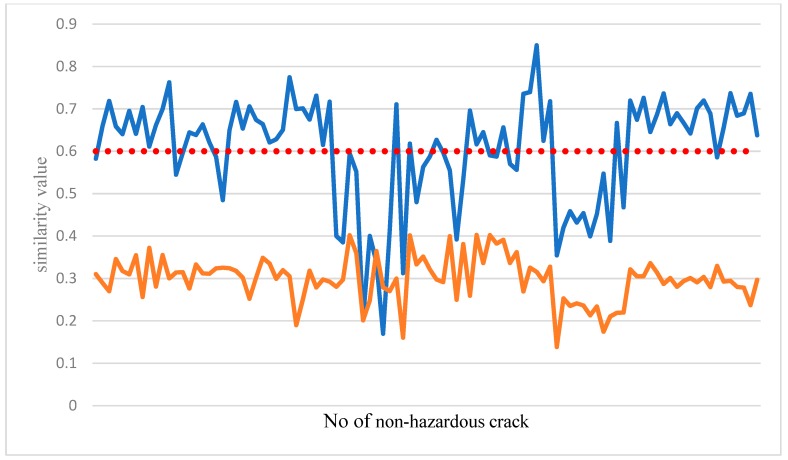
Joint feature comparison classifier result curve (blue: Joint; red: dangerous crack).

The joint feature comparison classifier was created by the sample in Figure 7. The horizontal axis range was between −1 and 1. Figure 8 compares the results of the non-dangerous crack feature comparison classifier. Most of the samples in the non-dangerous crack are flat. Therefore, when a dangerous crack enters a joint for classification, as in the coffee curve in Figure 8, all the similarity values are generally low, and vice versa, because the crack image and the sample edge in the sample database are tested. It was relatively close, so most of the blue curves in Figure 8 exceed the set threshold. So as long as the two test cases can be distinguished and the similarity value exceeds the threshold, the classification is successfully completed.

**Figure 9:**
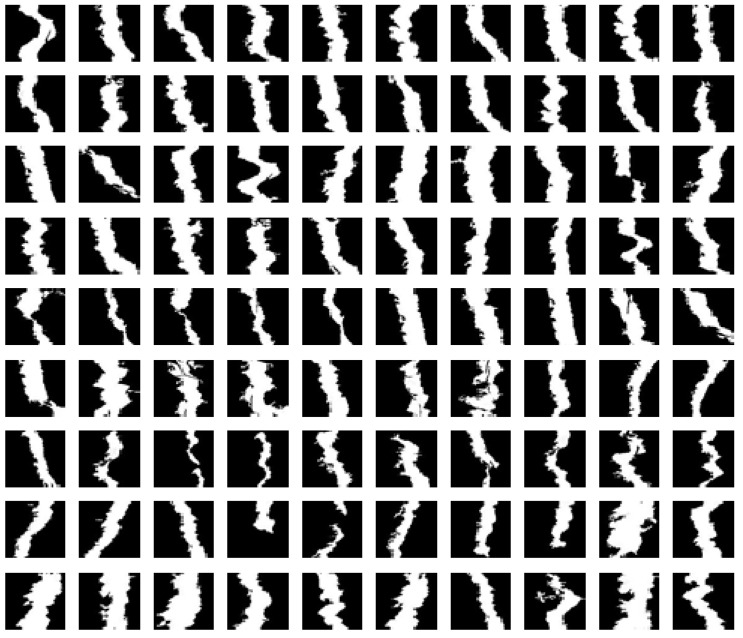
Dangerous crack sample database.

The samples in Figure 9 are also of a dangerous crack selected from 1000 positive samples, and the selected dangerous cracks are sequentially processed for image pre-processing, including morphological processing, in order to filter out other noises than cracks. At the same time, the pits in the crack are filled up, so that the crack can present a complete edge, and the probability of mismatching the result of the similarity calculation when the classifier compares the classifiers can be avoided. Finally, each A sample is unified to a size of 100 × 100.

**Figure 10:**
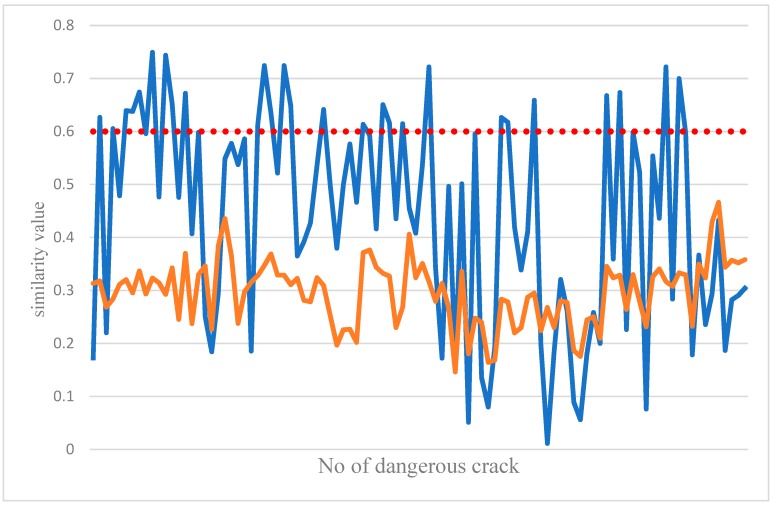
Curve of the classification results of the dangerous crack characteristics’ comparison classifier (red: Joint; blue: dangerous crack).

In the process of the feature comparison classifier, the most important thing is to set a stable threshold. This value can completely distinguish the classification results. Figure 10 shows the dangerous crack characteristics created by the classifier against dangerous cracks. And for the similarity value calculated when the dangerous crack is classified, the image of the test will be similar to the value of each dangerous crack in the dangerous crack sample. The blue curve in Figure 10 indicates the test image for the dangerous sample. In the image similarity value, the test result of the joint image is the brown curve in Figure 10. Since the edge of the crack is irregular, the crack of the test image will be similar to the image in the sample. The edge of the drop, rather than the dangerous crack, it is relatively flat, so the similarity value in the graph is relatively stable. From the graph, it can be judged how to set the threshold, so the current system similarity threshold is 0.6, so as long as the comparison between the image of the test and a sample in the sample has a threshold of 0.6, indicating the classification result, but it cannot guarantee each dangerous crack. The graphs will be such that there must be one size and one small size. It is also possible that the crack values tested were lower than the total similarity values in the sample. Therefore, in the sample database, each angle must be considered, and the sample should be considered as much as possible. The database has more different cracks to make the classification results more stable.

## 5. Conclusions

In this study, there was a good result in the crack detection of the integrated learning classifier. In the cascade classifier, as the number of positive and negative samples increases, a good classification result can be achieved. The feature comparison classifier is cascading. The classifier has 96% precision and can automatically compare the target area range.

Before the sample is prepared, each image must be pre-image processed, including the conversion of color space, grayscale and binarization processing, and then the pattern is processed according to the present sample state, which will obviously be miscellaneous. The purpose of the local removal is to allow the sample to have only clean cracks, to avoid the noise from affecting the alignment process, and finally, to perform the feature extraction of the Canny edge, and to further extract the preliminary crack location classified by the cascade classifier. For the edge feature, the sample alignment is performed by the target region’s edge feature and the sample’s edge feature re-feature comparison classifier, and the first feature comparison classifier is used to determine whether it is a non-dangerous crack. In the case of a dangerous crack, it is then judged by the characteristic comparison classifier, whether it is a dangerous crack.

In the feature comparison classifier, it is now possible to further determine whether a crack is dangerous, to screen all the classified cracks, and to cooperate with those who have background knowledge related to water conservancy or civil engineering to determine whether the dangerous crack presents immediate danger. The system can also pick out which cracks need to be tracked regularly, so that construction workers can work efficiently.

## Figures and Tables

**Table 1:** Cascade classifier in the rapeseed classification result data table.

Positive Sample	Negative Sample	Test Image	Unable to Judge	Classification Result	Correct Result	Accuracy (%)
200	400	100	32	68	40	58.82%
500	1000	100	7	93	81	87.09%
1000	2000	100	0	100	96	96%
